# Frequency and the Type of Chromosomal Abnormalities in Patients with Primary Amenorrhea in Northeast of Iran

**Published:** 2013-04

**Authors:** Farnaz Mohajertehran, Kazem Ghodsi, Leili Hafizi, Ameneh Rezaee

**Affiliations:** 1 Department of Genetics, Ghaem Hospital, Mashhad University of Medical Sciences , Mashhad, Iran; 2 Departmen of Medical Genetics and Immunology and Allergy Immunology and stem cell Department, Ghaem Hospital, Mashhad University of Medical Sciences, Mashhad, Iran.; 3 Obstetrics & Gynecology Department, Imam Reza Hospital, Mashhad University of Medical Sciences, Mashhad, Iran; 4 Urgency Department, Imam Reza Hospital, Mashhad University of Medical Sciences , Mashhad, Iran Dental Research Center,; 5 School of Dentistry, Mashhad University of Medical Sciences, Mashhad, Iran

**Keywords:** Chromosomal abnormalities, Cytogenetic Study, Iran, Karyotyping, Primary Amenorrhea

## Abstract

***Objective(s):*** Primary and secondary amenorrhea are different from each other in that the former refers to a physiological failure in the onset of spontaneous menarche during the time when it is expected. whereas the latter involves the cessation of normal menstruation any time prior to menopause. In this study we aimed to investigate chromosomal abnormalities in patients with Primary Amenorrhea in Northeast of Iran by employing GTG banding.

***Materials and Methods:*** Chromosomal analysis was carried out on 180 cases that were referred from different clinics in eastern cities of Iran to our laboratory from 2004 to 2009. We implemented the suggested protocol regarding peripheral blood lymphocyte culture for metaphase chromosome preparation as well as conventional analysis for G-banded chromosome.

***Results:*** The karyotype results revealed that 75.55% (n=136) had normal chromosome composition and 24.45% (n=44) showed chromosomal abnormalities. Among the patients with abnormal chromosome constituents 86.36% exhibit numerical aberration and 13.63% showed structural abnormalities. The most frequent abnormality detected was X chromosome monosomy, homogeneous (21 cases –11.66%) or mosaic (8 cases – 4.44%). The other 6 cases (3.33%) had X chromosome structural imbalanced abnormalities (homogeneous or in mosaic).

***Discussion:*** As expected, this study confirmed previously reported cytogentic abnormalities in patients with amenorrhea. Although there are percentage differences between these studies and also verities in chromosomal abnormalities, they have still demonstrated the importance of cytogenetic investigations in the etiological diagnosis of amenorrhea.

## Introduction

Amenorrhea is the absence or abnormal ending of the menses (1). Amenorrhea is defined as no menstruation by the age of 14 in the absence of growth or progress of secondary sexual characteristics; no menstruation by the age of 16 despite the presence of normal growth and progress with the appearance of secondary sexual characteristics; and in a woman who has been menstruating, the absence of menstruation for a length of time equivalent to a total of at least 3 of the previous cycle intervals, or 6 months of amenorrhea (2).

It is classified as primary amenorrhea (PA), which is the collapse menses by the age of 16, or secondary amenorrhea (SA), in which the menses appears at puberty but is subsequently ceased (1). The prevalence of PA in the United States is less than 1% and the occurrence of SA is 5-7%; no evidence indicates that the occurrence of amenorrhea varies according to national origin or ethnic group (3).

PA is primarily caused by: pituitary / hypothalamic disorders (27.8 %); gonadal dysfunction (50.4%); and outflow tract abnormalities (21.8 %) (1). As it can be observed, gonadal / ovarian disorders make up half of the total PA cases. This category of etiology often roots from abnormal sex chromosomes (3). 

Amenorrhea is a normal feature in pre pubertal, pregnant, and postmenopausal females and it accounts for 20% of infertility patients. The diagnosis disparity of amenorrhea is wide and can vary from endocrine disorders, genetic abnormalities, psychological, environmental, and structural anomalies. 

Thus karyotyping is one of the standard diagnostic procedures for identifying chromosomal abnormalities involved in this disorder (4-6).

The division of chromosomal abnormalities reported is greatly variable, from 15.9% to 63.3% for primary amenorrhea (7-13). 

We carried out a retrospective study, with the purpose of establishing the frequency and the type of chromosomal abnormalities, in 180 patients with PA who were referred to our genetics clinic and cytogenetics laboratory in Ghaem Hospital, Mashhad, Iran, 2004-2009. 

## Materials and Methods

The study subjects included patients with primary amenorrhea referred (n= 180) for chromosomal analysis to the Cytogenetic Laboratory of Ghaem Hospital, Mashhad University of Medical Sciences, Mashhad, Iran. The cases were referred from different clinics of the Khorasan province (North East of Iran) between 2004 and 2009.

The age group of the subjects ranged from 14 to 33 years with a mean of 21.7±5.1 years. Pedigrees with details were drawn and in depth clinical evaluation and clinical information were obtained from all subjects. 

Primary amenorrhea was described as the absence of menstruation and secondary sexual characteristics in phenotypic women aged 14 years or older or aged 16 or older if secondary sexual characteristics were present.

The diagnosis of primary amenorrhea was determined at the patient’s first visit and physical examination was performed to distinguish any secondary sexual characteristics or syndrome features. Laboratory examination and clinical information were obtained from hospital records or the referring physician.

All patients provided informed voluntary approval to participate in the study according to the protocol approved by the local Ethics Committee of MUMS.

About 1 ml of blood was supplemented with 8 ml of RPMI medium, 2 ml of fetal bovine serum, 0.1μg/ml of PHA (Phytohemaglutinin) and incubated at 37°C with 5% CO_2_. After 66 hour of incubation, ethidium bromide (1mg/ml) was added followed by the Colchicine (1mg/ml) at the 67th hour and incubated for another 1.5 hour (14). We obtained cells by applying a hypotonic solution of 0.075M KCl at 37°C for 20 minutes. This was followed by fixation and rinsing three times, using Carnoy’s fixative (methanol and acetic acid in a ratio of 3:1). The final outcome was then casted on clean slides, already cooled (15).

Twenty five metaphase spreads were investigated for each case, and when mosaicism was suspected, at least 50 metaphases were inspected. In order to identify the karyotypes, we took shots of the most revealing metaphases. In case of a translocation or any other abnormality, parents and siblings were also investigated. karyotypic reports were based on the International System for Human Cytogenetic Nomenclature recommendations (ISCN, 2009).

The relative frequency of each diagnostic group was addressed, and the percentage of abnormal cases and the division of the numerical and structural abnormalities were determined in each group. The frequencies were compared to similar studies using the Z-test for comparison of two frequencies with unequal variance.

## Results

**Figure 1.**
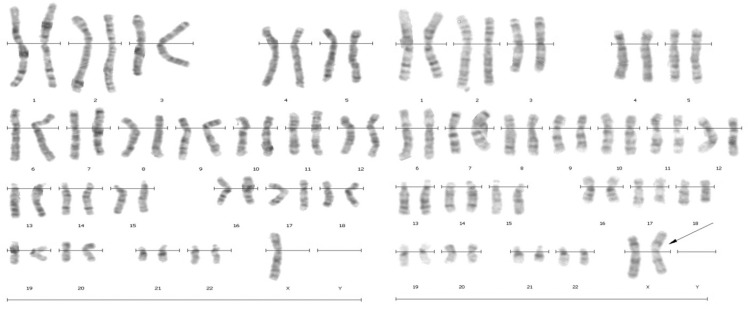
G-banded karyotype of patient with 45, X/46, X,iXq

The chromosomal analysis and karyotypes for the patients are summarized in table 1. The karyotype results showed 75.55% with normal chromosome composition (n=136) and 24.45% (n=44) demonstrated chromosomal abnormalities (Figure 1). The karyotype was normal in 136 cases (75.55%), with the PA having an etiology other than chromosomal abnormalities (hypothalamic or pituitary disorders, utero-vaginal abnormalities). 

 We identified a X chromosome homogeneous monosomy, 45, X in 21 cases (11.7%), a X chromosome monosomy mosaicism in 10 cases (5.6%), and other anomalies in 14 cases (7.8%) (Table 1).

In 13 cases (7.2%) we discovered a mosaic aneuploidy having mostly the 45, X cell line. We found 2 cases (1.2%) with mosaicism with 1 cell line with X monosomy and the second line with an unbalanced structural abnormality of X chromosome: including isochromosomes Xq (1 case- 0.56%), and ring X chromosomes (1 case– 0.56%) (Table 1 and Figure 1).

## Discussion

It is now clear that genetic disorders could have a considerable health and economic impact on affected individuals, families, and their society. As indicated from previous studies genetic causes of amenorrhea account for approximately 45% of cases—which may be a result of, for example, gonadal dysgenesis, chromosomal disorders, or Müllerian agenesis (14). Several studies have previously been carried out to determine the frequency of sex-chromosome abnormalities among patients with primary amenorrhea (15-17). It has been showen that chromosomal abnormalities are present in 46%–62% of patients with primary amenorrhea including X aneuploidy, male karyotype, or structural X-chromosome abnormalities which could be presented as X isochromosome, isodicentrics, rings, and deleted or inverted X chromosomes (18).

**Table 1:** Karyotype particulars of the subjects referred for cytogenetic analysis with primary amenorrhea. (n=180)

Chromosomal abnormalities	Karyotype	Number of cases	Frequency (%)
Normal Karyotype	46,XX	136	75.5%
Numerical Abnormality			
Monosomy X	45,X	21	11.7%
Sex reversal	46,XY	08	4.4%
Turners Mosaic	45,X/46,XX	07	3.9%
Presence of XY constitution	46,XX/46,XY 45,X/46,XY	02	1.1%
Structural Abnormality			
Isochromosome	46,X,iXq	04	2.3%
Mosaic structural X	45,X/46,X,i(Xq)	01	0.5%
Mosaic unbalanced structural X	45,X/46,X,r(X)	01	0.5%

In the present study the high percentage of chromosomal abnormalities (24.44%) detected in our patients with PA suggests their major role in the abnormal gonadal development and function as previously reported. Abnormal karyotypes incidence has been claimed to be greatly variable, ranging from 15.9% to 63.3% in women with a diagnosis of primary amenorrhea (Wong and Lam, 2005) (7), with the majority falling between 24% and 46% (Cortes - Gutierrez et al 2007) (18). Our result (24.44%) was in accordance to the results obtained by Wong and Lam, 2005,(24.50%) (7), Vijayalaksmi et al 2010 (27.8%) (15), Kalavathi et al 2010 (25.82%) (19), Ramirez et al 2000 (36.7%) (20) and Safaei et al 2010 (20%) (13) and lower than other studies: Kong et al 2007 (58.8%) (21) and Butnariu et al 2011 (54.56%) (22) (Table 2). The differences between the results of various studies may be due to the wide variation in patient origin in different studies. Male karyotype was observed in a significant percentage of patients with primary amenorrhea in previous studies, ranging from 3.3% to 13.7% (Table 2) and our study is comparable with these studies.

**Table 2:** Chromosomal abnormalities identified in cases with primary amenorrhea in different studies

Karyotype results	Present study	Wong et al (7)	Kong et al (21)	Vijayalaksmi et al (15)	Kalavathi et al. (19)	Ramirez et al (20)	Safaei et al (13 )	Butnariu et al (22)	Ten et al (9)
Frequency of abnormal Karyotype (number of cases)	24.44% (44)	24.5% (58)	58.8% (10)	27.8% (39)	25.82% (220)	36.7% (96)	20% (44)	54.56% (269)	30.8% (36)
X chromosome (homogeneous/mosaics) aneuploidies *	63.6% (28)	50% (29)	20% (2)	45.45% (100)	45.45% (100)	89.58% (92)	52.27% (23)	82.15% (221)	7.7% (9)
X chromosome unbalanced structural abnormalities	9.1% (4)	12.06% (7)	50% (5)	27.27% (60)	27.27% (60	4.16% (4)	15.90% (7)	8.17% (22)	-
Marker chromosome	-	1.72% (1)	-	-	-	-		-	1.7% (2)
Mosaics X/XY and variants	9.1% (4)	1.72% (1)	-	-	3.63% (8)	-	4.54% (2)	3.34% (9)	-
46,XY	18.2% (8)	8.4% (20)	30% (3)	17.9% (7)	23.63% (52)	7.85% (3)	27.27% (12)	5.20 % (14)	13.7% (16)
Other anomalies	-			-	7.23%	-		-1.11% (3)	

*were included 45,X and 47,XXX- -homogeneous and mosaics

## Conclusion

Chromosomal abnormalities demonstrated by various studies including the present investigation suggest that we should aim to continue the study of PA with more samples from other provinces of Iran to have a more accurate understanding of specific chromosomal abnormalities involved in PA in our country. Based on the above results we recommend that genetic counseling should be advised for marriage of couples with family history and attention should be paid to the risks of premature menopause for patients with Turner’s syndrome and also the use of hormonal replacement therapy should be recommended.
